# Estimating severe fever with thrombocytopenia syndrome transmission using machine learning methods in South Korea

**DOI:** 10.1038/s41598-021-01361-9

**Published:** 2021-11-08

**Authors:** Giphil Cho, Seungheon Lee, Hyojung Lee

**Affiliations:** 1 Finance·Fishery·Manufacture Industrial Mathematics Center on Big Data, Pusan National University, Busan, 46241 Korea; 2 Department of Mathematics, Pusan National University, Busan, 46241 Korea; 3 Department of Statistics, Kyungpook National University, Daegu, 41566 Korea

**Keywords:** Machine learning, Viral infection

## Abstract

Severe fever with thrombocytopenia syndrome (SFTS) is an emerging tick-borne infectious disease in China, Japan, and Korea. This study aimed to estimate the monthly SFTS occurrence and the monthly number of SFTS cases in the geographical area in Korea using epidemiological data including demographic, geographic, and meteorological factors. Important features were chosen through univariate feature selection. Two models using machine learning methods were analyzed: the classification model in machine learning (CMML) and regression model in machine learning (RMML). We developed a novel model incorporating the CMML results into RMML, defined as modified-RMML. Feature importance was computed to assess the contribution of estimating the number of SFTS cases using modified-RMML. Aspect to the accuracy of the novel model, the performance of modified-RMML was improved by reducing the MSE for the test data as 12.6–52.2%, compared to the RMML using five machine learning methods. During the period of increasing the SFTS cases from May to October, the modified-RMML could give more accurate estimation. Computing the feature importance, it is clearly observed that climate factors such as average maximum temperature, precipitation as well as mountain visitors, and the estimation of SFTS occurrence obtained from CMML had high Gini importance. The novel model incorporating CMML and RMML models improves the accuracy of the estimation of SFTS cases. Using the model, climate factors, including temperature, relative humidity, and mountain visitors play important roles in transmitting SFTS in Korea. Our findings highlighted that the guidelines for mountain visitors to prevent SFTS transmissions should be addressed. Moreover, it provides important insights for establishing control interventions that predict early identification of SFTS cases.

## Introduction

Severe fever with thrombocytopenia syndrome (SFTS) is an emerging tick-borne infectious disease in China, Japan, and Korea, caused by a novel bunyavirus SFTS virus (SFTSV) belonging to the *Phlebovirus* genus. SFTS was listed as one of the nine most infectious diseases in the World Health Organization priority list in 2017^1^. SFTS is an increasingly important threat to public health because it is an infectious disease with a high fatality rate, and the number of countries affected with SFTS has increased.

SFTS is characterized by high fever, thrombocytopenia, leukopenia, gastrointestinal symptoms (vomiting and diarrhea), hemorrhage, and multiorgan dysfunction^2^. The incubation period of SFTS is generally 6–14 days, with an average of 9 days^3^. The average case fatality rate varies from 8 to 12%, but it can be as high as 30%^4^. SFTS cases were first reported in China in 2009 and in Korea and Japan in 2013^5^. SFTSV has been detected in several species of ticks, including *Haemaphysalis longicornis*, *Amblyomma testudinarium*, and *Ixodes nipponensis*. SFTSV has been detected in various reservoir animal species including domestic animals such as cats, mice, and wild boars and has been incidentally detected in humans^6^. Although there is neither a specific vaccine nor antiviral treatment for SFTS, SFTSV has a wide distribution, and epidemic areas of SFTS have been expanding^7^; therefore, it poses a significant threat to global health.

Previous studies have reported that meteorological factors influence the risk of tick-borne infections by affecting tick growth dynamics, tick-human interactions, and virus replication^8^. Meteorological factors, including temperature, relative humidity, precipitation, duration of sunshine, and environmental factors, such as land cover and tick density, have been associated with the occurrence of SFTS^9^. The disease usually presents from March to November, peaking between May and July. Moreover, SFTS cases are frequently identified among farmers residing in rural regions or near forested or wooded and hilly areas^10^.

Several previous studies have reported risk factors of SFTS infection analyzed using epidemiological data^9–14^. The risk factors of SFTS cases in China have been analyzed using univariate analysis of epidemiological data^11,13^. Moreover, previous studies have estimated the occurrence of SFTS by assessing risk factors^10,12,14^. The risk of demographic and spatiotemporal features of SFTS occurrence in Shandong Province has been estimated using the maximum entropy niche model^14^. Comprehensive epidemiological characteristics have been described to assess the risk of human SFTS infection in China^10^ using a boosted regression tree (BRT) model, considering climate conditions and forest coverage. The potential risk of the geographical distribution of *H. longicornis* was estimated using the BRT model^12^. The distribution of tick species, including *H. longicornis* in Korea, has been investigated previously^15^. Gaff and Gross used mathematical modeling to evaluate strategies for predicting and managing outbreaks of tick-borne diseases^16^. The number of domestic animals infected by the SFTSV was estimated using the SIR model in Shandong, China^17^.

The first SFTS patient was reported in Korea in 2013. Between 2013 and 2019, a total of 1089 confirmed SFTS cases and 214 deaths were reported in Korea^18^. The regions in Korea included seven geographical areas: the Seoul Metropolitan Area, Chungcheong area, Gyeongbuk area, Gyeongnam area, Honam area, Gangwon-do, and Jeju. The numbers of SFTS cases between 2013 and 2019 are summarized in Supplementary Table S1. Meteorological data relative to average temperature and precipitation were compared with the number of SFTS cases. As shown in Supplementary Fig. S1, meteorological factors play important roles in the dynamics of the number of SFTS cases. A heterogeneity of the spatiotemporal transmission of SFTS has been observed in recent years. A large number of SFTS cases have been reported in Gyeonggi-do, Gangwon-do, and Gyeongsangbuk-do in Korea from 2016 to 2019. Due to climate changes (global warming), the potential risk of SFTS infection has increased in Korea, which has gradually transformed into a subtropical region.

In this study, our objective was to estimate the occurrence of SFTS and the number of SFTS cases in geographical areas in Korea, based on meteorological and environmental factors. First, we identified the main modes of SFTS transmission at the regional level. Second, the potential risk of SFTS infection was estimated to determine whether an SFTS outbreak had occurred in each area. Finally, the number of SFTS cases was estimated using machine learning methods.

## Results

### General epidemiological characteristics

**Figure 1:**
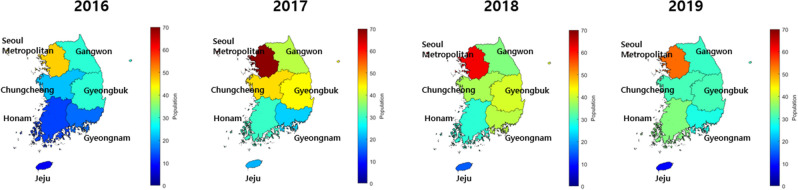
Distribution of the number of SFTS cases stratified by seven geographical areas from 2016 to 2019 in Korea. The map is simply drawn using MATLAB R2020b from GIS shape file (http://www.gisdeveloper.co.kr/).

Epidemiological characteristics, including demographic, geographic, and meteorological categories, are summarized in Supplementary Table S2. A total of 913 SFTS cases, including 160 fatal cases, were reported from 2016 to 2019 in Korea. The average case fatality rate (CFR) was 17.41%, including 50 males (CFR: 16.34%) and 50 females (CFR: 18.48%). A total of 165, 272, 259, and 223 SFTS cases were reported each year. The year with the highest number of SFTS cases was 2017 (29.63%), and the year with the least number of cases was 2016 (17.97%). The seven geographical areas are shown in Fig. 1. The capital area had the highest number of cases (25.27%), and the Jeju area reported the least number of cases (5.77%) among the seven geographical areas from 2016 to 2019. The SFTS cases revealed a seasonal pattern, with 91.73% of cases occurring between June and October. The highest number of cases was reported in October, whereas no cases were reported in January and December in each year.

According to geographic characteristics, the land area in Korea is 99,900 km^2^, and Gangwon includes a relatively large forest (81.82%) with respect to the land area. The geographical area with the highest rate of farmers was the Honam area (0.17). Gangwon had the highest altitude (525 m), whereas the capital area had the lowest altitude (481 m). Information relative to a total of 2,749 mountain visitors who traveled to national parks was collected between 2016 and 2019. The Gyeongbuk and Honam areas included the most mountain visitors (43.69%). Meteorological characteristics showed that the average temperature, average maximum temperature, average minimum temperature, average relative humidity, precipitation, and average percentage of sunshine were 13.80 °C/month, 18.88 °C/month, 9.40 °C/month, 67.61%/month, 106.08 mm/month, and 53.64%/month, respectively.

### Feature selection

The results of the univariate feature selection are shown in Supplementary Table S3 according to the CMML or RMML, respectively, among the 15 features. We selected all features that were revealed to be important features with having a *P*-value < 0.05, for either CMML or RMML. As a result, a total of nine features among the 15 evaluated features were selected (Supplementary Fig. S2) and included month, average temperature, average maximum temperature, average minimum temperature, average relative humidity, precipitation, average percentage of sunshine, number of mountain visitors, and population. Comparing important features between the CMML and RMML models, the population feature was statistically significant in the RMML but not in the CMML model. In addition, two features, month and mountain visitors, displayed relatively higher RMML scores than those from CMML, which indicated that the two features were significantly more significant in RMML.

A correlation analysis was performed between the nine selected features and SFTS occurrence (Supplementary Fig. S3A). There was a negative correlation between SFTS occurrence and the average percentage of sunshine with a correlation coefficient of − 0.32. and a positive correlation of SFTS occurrence with three features of temperature (0.63–0.64) and relative humidity (0.56). Similarly, Supplementary Fig. S3B shows that the number of SFTS cases had a negative correlation with the average percentage of sunshine (− 0.19), whereas it was positively correlated with the temperatures (0.46–0.47) and relative humidity (0.41). Moreover, we observed that the average temperature, average maximum temperature, and average minimum temperature were highly correlated, with correlation coefficients higher than 0.98. The average maximum temperature was only included as an important feature because it showed the highest score among the three temperature features according to the univariate feature selection (see Supplementary Table S3). Finally, we obtained seven important features, excluding the average temperature and average minimum temperature of the nine selected features.

### Estimation of SFTS occurrence

**Figure 2:**
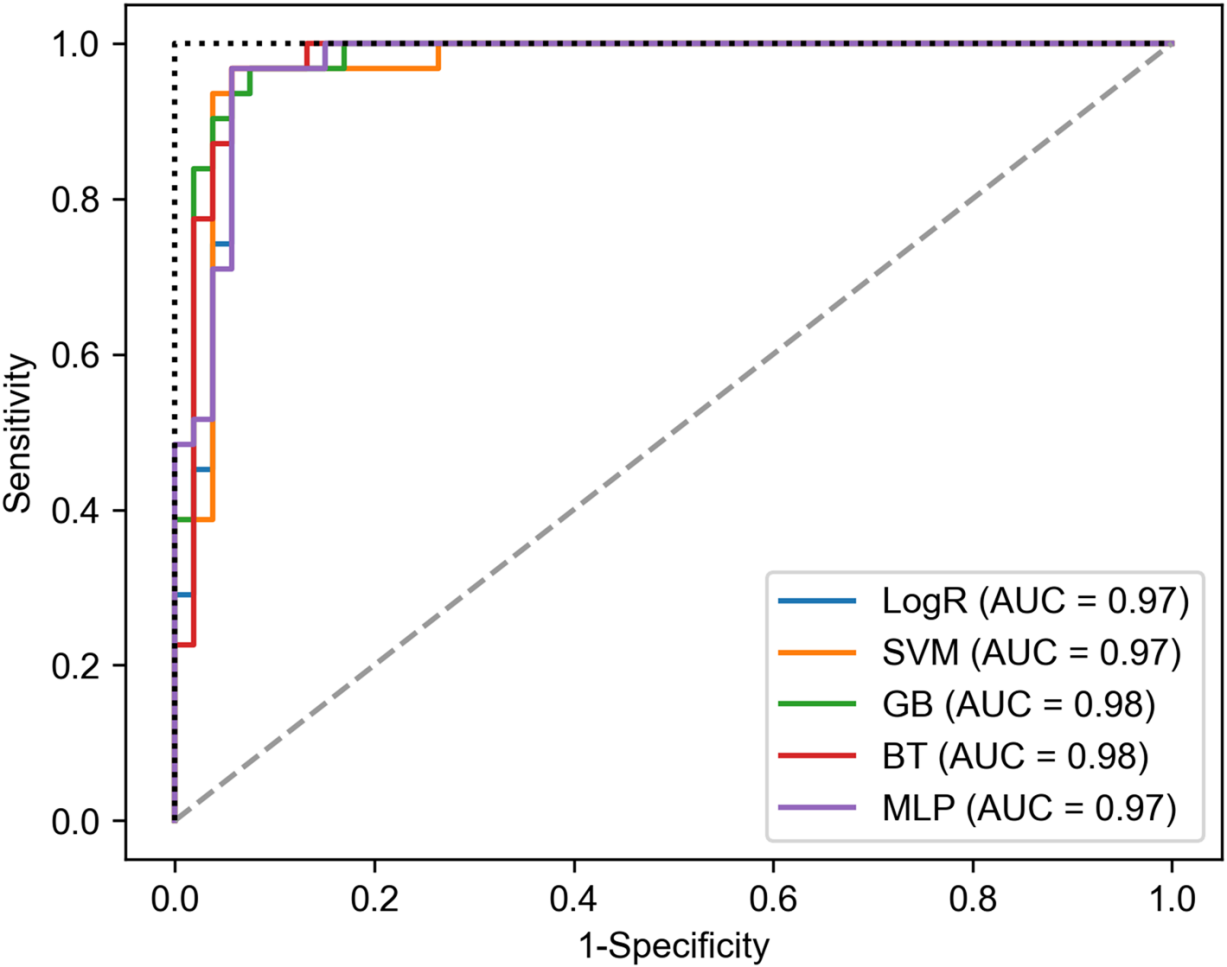
The receiver operating characteristic curve comparing five classification models in machine learning.

Epidemiological data in seven geographical areas were used for training data from 2016 to 2018 and testing data in 2019, employing five machine learning methods. The test data were set as the SFTS cases to estimate the SFTS occurrence in seven geographical areas, adhering to the threshold of occurrence as three cases of SFTS. Table 1 shows the performance with the mean and 95% confidence intervals (95% CIs) of 100 simulations of training and test data. All results of the test data had high accuracy (higher than 90%), whereas the results of the training data showed 83–100% accuracy. For the prevalence rate of the data, the train data includes 90 positive cases among a total of 252 cases (90/252 (33.3%)). The test data includes 31 positive cases among 84 cases (31/84 (34.4%)). Therefore, the prevalence rates of train data and test data were not significantly different. In addition, GB and BT methods showed higher accuracy, with values above 90% for both the test data and training data, which indicated that both GB and BT were the most suitable machine learning methods, with respect to the high accuracy. Figure 2 shows the results from five different machine learning methods evaluated by the receiver operating characteristic curve with area under the curve (AUC). The AUCs of the GB and BT methods were 0.959–0.991, indicating an excellent prediction method for SFTS occurrence. Moreover, performance was compared according to the five machine learning methods using several thresholds (1, 2, 3, 4, and 5) to define SFTS occurrence as shown in Supplementary Table S4. The results indicated that a threshold of three cases provided the most accurate result for CMML estimation of SFTS occurrence.

**Table 1:** Performance of train and test data of estimating severe fever with thrombocytopenia syndrome occurrence, using the classification model in machine learning.

Estimator	Training cases (*n* = 252)			Test cases (*n* = 84)		
	Accuracy	F1-score	AUC	Accuracy	F1-score	AUC
LogR	0.833	0.767	0.917	0.917	0.885	0.968
SVM	0.893	0.862	0.938	0.940	0.923	0.969
GB	1.000 (1.000, 1.000)	1.000 (1.000, 1.000)	1.000 (1.000, 1.000)	0.937 (0.929, 0.940)	0.915 (0.903, 0.921)	0.977 (0.972, 0.983)
BT	0.948 (0.933, 0.964)	0.930 (0.911, 0.952)	0.992 (0.986, 0.996)	0.936 (0.917, 0.952)	0.913 (0.885, 0.938)	0.978 (0.959, 0.991)
MLP	0.859 (0.847, 0.869)	0.812 (0.789, 0.827)	0.920 (0.915, 0.926)	0.940 (0.917, 0.952)	0.921 (0.892, 0.938)	0.970 (0.965, 0.974)

Numbers in parentheses indicate the 95% confidence interval obtained from 100 simulations.

### Estimation of the number of SFTS cases using regression model in machine learning (RMML)

We fit the data of seven geographical areas to estimate the number of SFTS cases and the cumulative number of SFTS cases. The comparison between the observed and estimated cases is shown in Supplementary Figs. S4–S7. Supplementary Table S5 compares the accuracy with respect to the mean squared error (MSE) and the coefficient determination (R^2^) using the five machine learning methods of RMML. GBR and BTR showed a lower MSE and higher R^2^ than those of the other three methods for the training data. In detail, according to BTR, R^2^ values for the training data and test data were 0.86 and 0.75, respectively, and the MSE values were similar for the training and test data. However, according to GBR, R^2^ values for the training data and test data were 0.98 and 0.73, respectively, whereas GBR had a significantly smaller MSE for the training data (MSE: 0.41) than the test data (MSE: 3.64). Therefore, it may be interpreted that BTR is the best method for estimating the number of SFTS cases considering stable performance regardless of the training and testing datasets.

**Figure 3:**
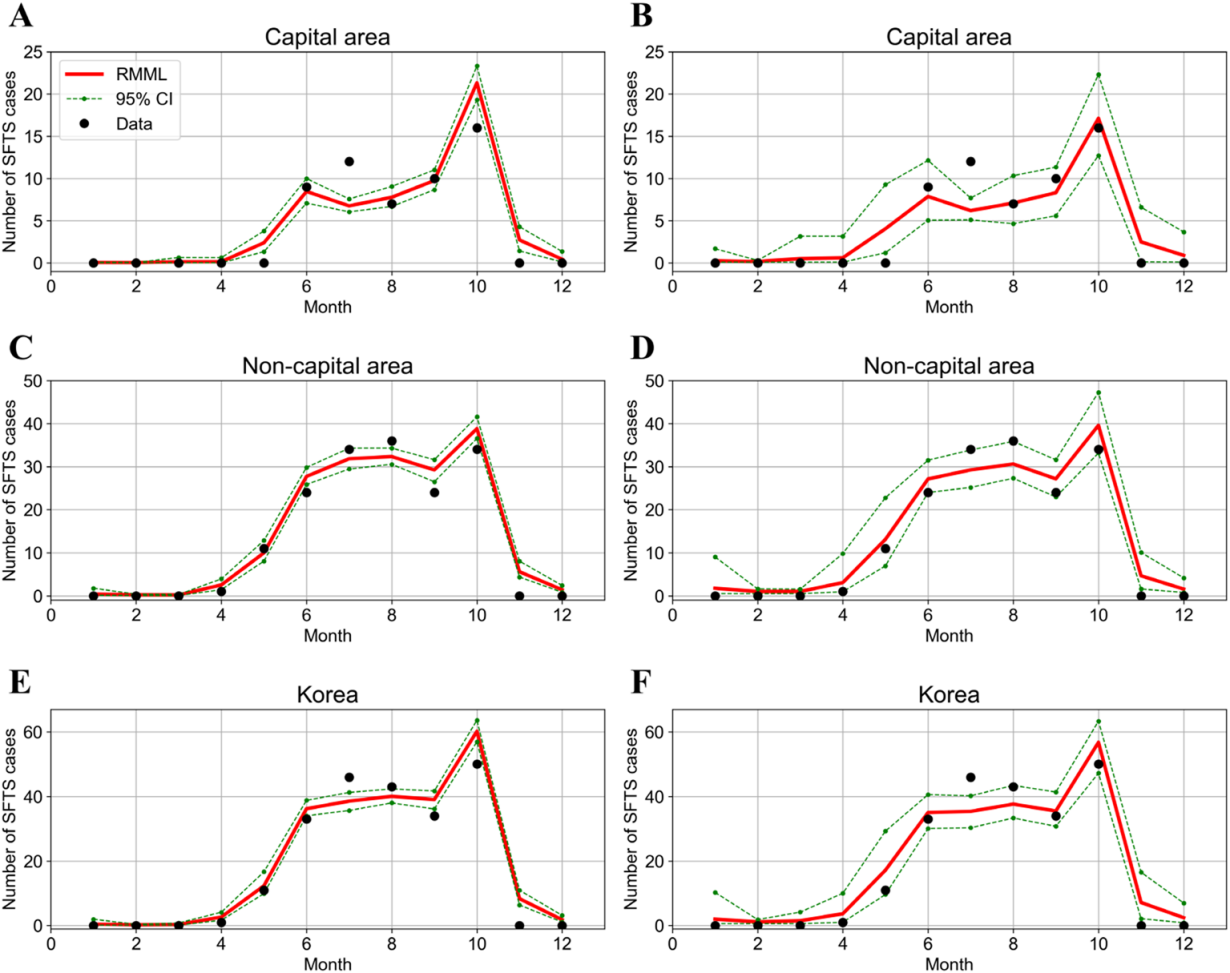
Comparison of observed cases and estimated values of the number of severe fever with thrombocytopenia syndrome (SFTS) cases. In (**A**–**B**), capital area; (**C**–**D**), non-capital area; and (**E**–**F**), Korea. The black dots represent the observed cases, and the red solid lines indicate the estimated values of SFTS cases with 95% confidence intervals shown in green dashed lines. Gradient boosting was employed in (**A**, **C**, and **E**), and the bagging tree method was used in (**B**, **D**, and **F**).

Here, to carefully compare incidence rates, we categorized three groups across seven geographical areas: Korea, capital area, and non-capital area. Figure 3 shows a comparison of the estimated and observed cases using GBR and BTR of RMML with 95% CIs in the capital area, non-capital area, and Korea. We observed that the models were well fitted to estimate the number of SFTS cases, except for the unexpectedly large number of SFTS cases reported in July.

### Estimation of the number of SFTS cases using the modified-RMML

The modified-RMML was used to improve the estimation of the number of SFTS cases. The comparison between the observed and estimated cases between RMML and modified-RMML is shown in Supplementary Figs. S8–S11. The modified-RMML was defined by adding two features into the RMML model identified from the outcomes of the best two models (GBR and BTR) of the classification model. Supplementary Fig. S12 and Table 2 show the performance of the modified-RMML with respect to MSE and R^2^. The performance improved using modified-RMML compared to the RMML using five machine learning methods by reducing the MSE of the training data (40.6%–79.2%) and of the test data (12.6%–52.2%).

**Table 2:** Performance of the training and test data of severe fever with thrombocytopenia syndrome cases using the five modified regression models in machine learning (modified-RMML).

Methods	Modified-RMML			
	Train		Test	
	MSE	R^2^	MSE	R^2^
LR	6.558 (− 40.6)	0.636 (64.5)	4.175 (− 35.3)	0.687 (33.0)
Ridge	6.562 (− 40.6)	0.636 (64.6)	4.162 (− 35.5)	0.689 (33.2)
GBR	0.187 (− 54.8)	0.990 (1.29)	3.127 (− 14.2)	0.766 (5.31)
BTR	1.373 (− 46.6)	0.924 (7.75)	2.937 (− 12.6)	0.780 (4.24)
MLPR	2.329 (− 79.2)	0.871 (130)	3.190 (− 52.2)	0.761 (52.0)

Parentheses represent the percentage decrease of MSE from the modified-RMML compared to the RMML.

There are two main reasons for choosing the best methods for modified-RMML: (i) MLPR had the highest improvement by reducing the MSE for both training (79.2%) and test data (52.2%), and (ii) BTR resulted in a low MSE and high R^2^ and a stable performance for training and testing datasets, which was similar to that of the RMML. The BTR and GBR of the modified-RMML showed low MSE and high R^2^ values. However, the MSE using the BTR was similar to that of the training and test data, whereas the MSE using GBR for the training data was significantly smaller than that for the test data. Therefore, we concluded that the BTR was also the best approach for the modified-RMML.

**Figure 4:**
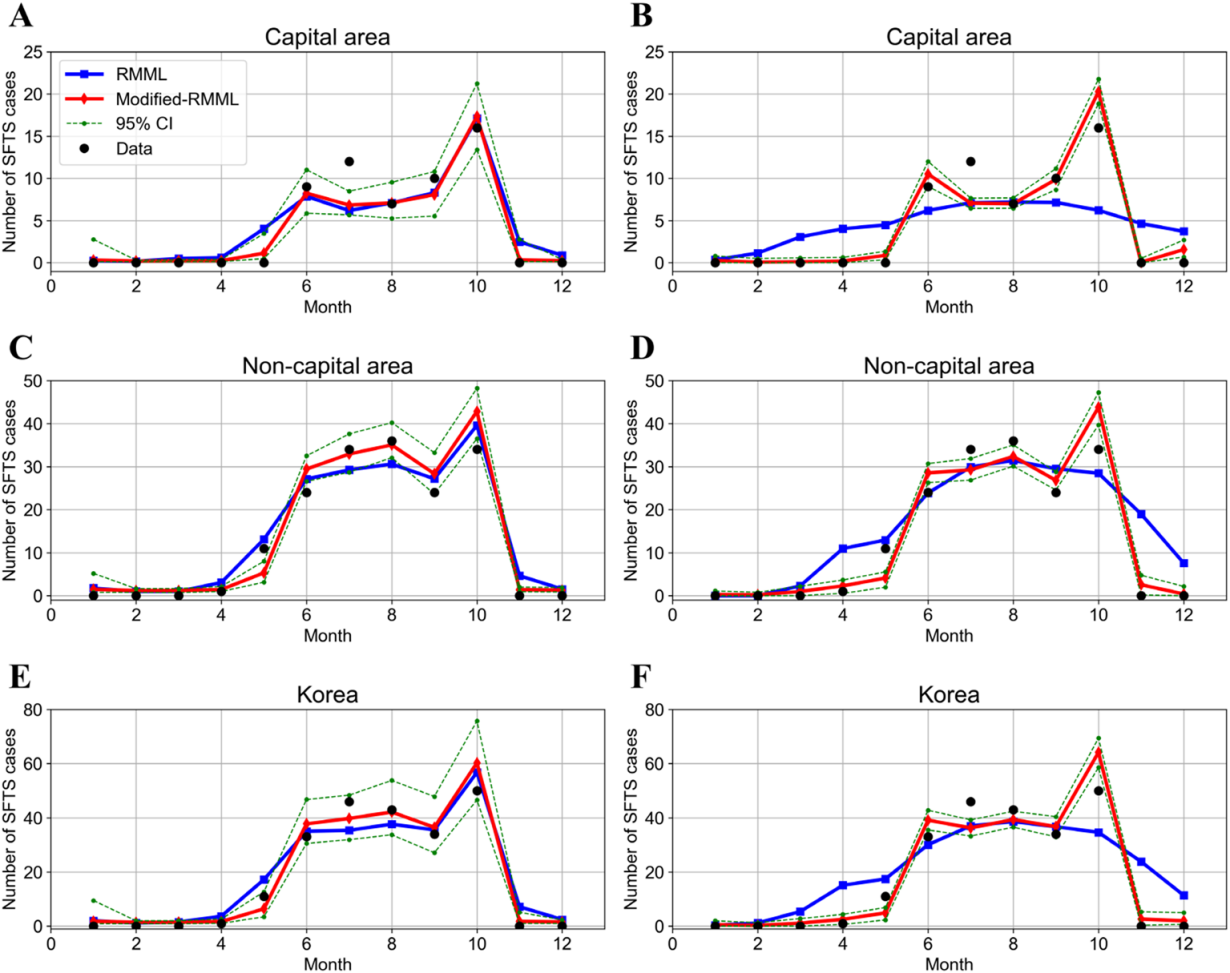
Comparison of the results of the regression model in machine learning (RMML) and modified regression models (modified-RMMLs) to estimate the number of severe fever with thrombocytopenia syndrome (SFTS) cases. Capital area (**A**–**B**), non-capital area (**C**–**D**), and Korea (**E**–**F**). The black dots represent the observed cases. The blue solid lines and red solid lines indicate the estimated SFTS cases from the RMML and modified-RMML, respectively. The 95% confidence intervals of the modified-RMML are shown in green dashed lines. The results of the two models using the bagging tree regression approach are shown in (**A**, **C**, and **E**), and the results obtained from the artificial neural network (multi-layer perceptron regression) are shown in (**B**, **D**, and **F**).

Figure 4 compares the observed and estimated cases using BTR (left panel) and MLPR (right panel) approach with their respective 95% CIs. The observed data reported in May and July were not suitably estimated using the RMML. Although the extraordinarily high values reported in July could not be estimated even using either the RMML or the modified-RMML, the observed data in May was successfully estimated using the MLPR of the modified-RMML.

**Figure 5:**
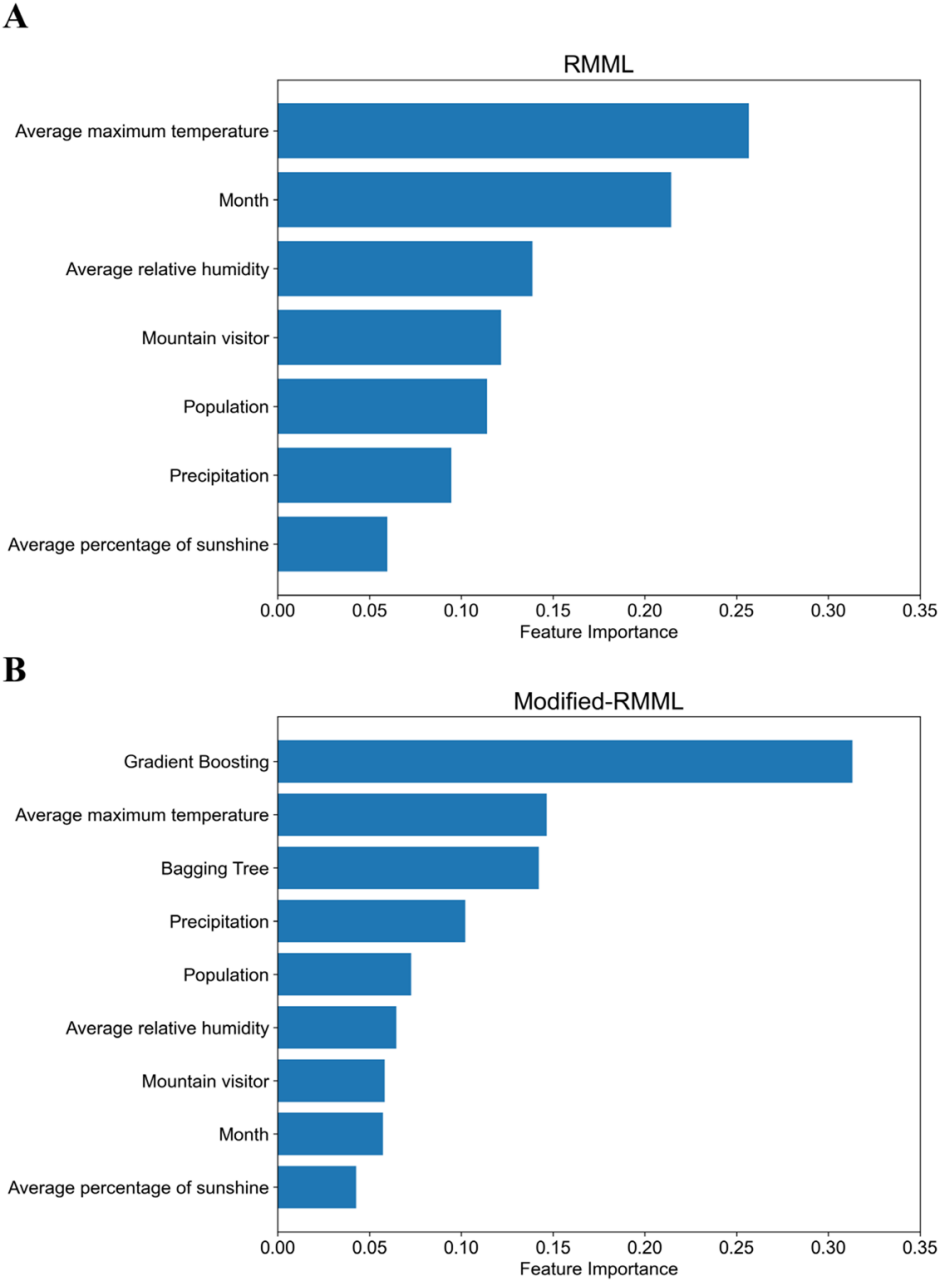
Comparison of the feature importance between gradient boosting method of the regression model in machine learning (RMML) (**A**) and modified-RMML (**B**).

Feature importance was analyzed using the GBR method for the RMML and the modified-RMML. In the RMML, the average maximum temperature was found to be the most important feature (Fig. 5A) and showed the highest score for univariate feature selection. The features of month, population, mountain visitors, and average relative humidity resulted to be important features with Gini values higher than 0.1 of Gini importance. As shown in the modified-RMML in Fig. 5B, the outcome obtained from the GB method of CMML and the average maximum temperature were the most important features with 0.31 and 0.15 of Gini importance, respectively. Moreover, considering the outcome from the BT method of CMML, precipitation also had high Gini importance, with values higher than 0.1.

## Discussion

In the present study, the epidemic characteristics of SFTS cases in Korea were elucidated, and environmental factors and meteorological factors were explored. Our study aimed to assess the risk factors for SFTS infection in Korea. We used three models to achieve the following two purposes: (i) to estimate the SFTS occurrence using CMML and (ii) to estimate the number of SFTS cases using RMML or the modified-RMML by employing machine learning methods. Herein, we used RMML, which is a well-known model, although we have proposed a modified-RMML approach in this study for the first time to estimate the number of SFTS cases. First, seven important features were selected through univariate feature selection and correlation analysis, including month, average maximum temperature, average relative humidity, precipitation, average percentage of sunshine, mountain visitors, and population (see Supplementary Fig. S2). Second, we predicted the SFTS occurrence using the CMML for the year 2019 using data from 2016 to 2018. A SFTS occurrence was defined if more than three SFTS cases were reported in a month. As a result, GB and BT were the two best methods, with accuracies higher than 90% for both the testing data and training data shown in Table 1. Regardless of the threshold values, the GB and BT showed good performances with high accuracy (see Supplementary Table S4).

Third, using RMML, the GBR and BTR had high R^2^ accuracies of 0.73 and 0.75, respectively, with respect to the test data. We concluded that the BTR was the best method for RMML because of the consistent accuracy for the training and test datasets. Fourth, we constructed a novel model, the modified-RMML, by incorporating the outcomes of the best two methods (i.e., GB and BT) for the CMML model into the RMML. The two models, RMML and modified-RMML, were compared with respect to their performance in predicting the number of SFTS cases in a geographical area. As shown in Fig. 4, the SFTS cases were better predicted using the modified-RMML. Table 2 shows that performance was improved using modified-RMML compared to RMML by reducing the MSE. MLPR showed the highest improvement by reducing the MSE for both the training (79.2%) and test datasets (52.2%). BTR was considered the best method of the modified-RMML to achieve lower MSE and higher R^2^ and was consistent MSE for training and testing datasets. Finally, we analyzed the feature importance using the modified-RMML. We found that the outcome obtained from the GB method of the CMML and the average maximum temperature were the most important features with 0.31 and 0.15 Gini importance, respectively.

In our study, we propose the novel modified-RMML model to predict the number of cases of SFTS, which improved the performance of the previously developed RMML. In addition, we found that this performance improved by incorporating the prediction for SFTS occurrence.

To the best of our knowledge, this is the first study to use a modified-RMML to predict the number of SFTS cases, which was determined by employing machine learning methods based on epidemiological characteristics such as climate factors, demographic factors, and geographical factors in Korea. In addition, we found that the performance improved by incorporating the estimation of SFTS occurrence. In particular, the performance using MLPR of the modified-RMML showed the highest improvement by reducing the MSE to 79.2% for the training data and 52.2% for the test data.

Previous studies^10,12^ have used the BRT model of the machine learning method to assess the risks of SFTS occurrence. Comparing AUC values used to estimate the occurrence of SFTS, the AUCs for the training and test datasets were higher than 0.9 using the BRT model^12^. Wang et al. have estimated the AUC values for the SFTS occurrence using temperature and precipitation at 0.79–0.91^14^ using the maximum entropy niche model. In the present study, we achieved 0.986–1.00 AUC values for the training dataset of the SFTS cases from 2016 to 2018 and 0.959–0.991 for test data of SFTS cases in 2019, using BT and GB machine learning methods of CMML.

The results of feature importance in the modified-RMML indicated that the outcomes of estimating the SFTS occurrence using CMML were the most important. Moreover, climate factors, including temperature and relative humidity, and geographical factors such as the number of mountain visitors played an important role in predicting the number of SFTS cases. Thus, we successfully estimated the number of SFTS in seven geographical areas in Korea using a novel model of modified-RMML.

The present study has several limitations. First, asymptomatic cases and underreporting cases of SFTS are inherent limitations of any epidemiological SFTS study. Mild and subclinical cases were not identified^10,19^. Next, it was difficult to obtain a sufficient number of suitable laboratory samples and construct a surveillance system to promptly identify SFTS cases. Second, considering the environmental factors, a detailed investigation was limited, which might have had an impact on the number of SFTS cases^10,14^. Finally, the transmissibility of the SFTSV is complicated as effective contact can occur between ticks and humans and other reservoir hosts^10^.

However, we incorporated the transmission dynamics of SFTS cases with climate factors and geographical factors that affect the distribution of tick populations. Third, the tick population was not included in our analysis due to the lack of available data for tick populations. Miao et al. have estimated the probability of the presence of ticks infected by the SFTSV^10^, and Liu et al. have estimated the occurrence of SFTS using binary data for the presence of *H. longicornis* ticks geographically^10^. In our study, this approach was not suitable as *H. longicornis* are the most commonly found tick species in Korea^15^.

## Conclusions

The present study estimated the occurrence rate and the number of SFTS cases using demographic, geographic, and meteorological factors. The results provide important insights for control interventions to be implemented by governments. Our model can provide an indicator for the early identification of SFTS occurrence and the information relative to high-risk areas of SFTS using the CMML model. In particular, mountain visitors and climate factors, such as temperature and relative humidity, play a critical role in SFTS transmission. During the period of increasing the SFTS cases from May to October, the modified-RMML could give more accurate estimation. Our findings underline the need to implement guidelines for mountain visitors to prevent SFTS transmissions. Moreover, this study was the first to propose the novel modified-RMML model to estimate the number of SFTS cases, which improved from the application of previously developed machine learning methods.

## Methods

**Figure 6:**
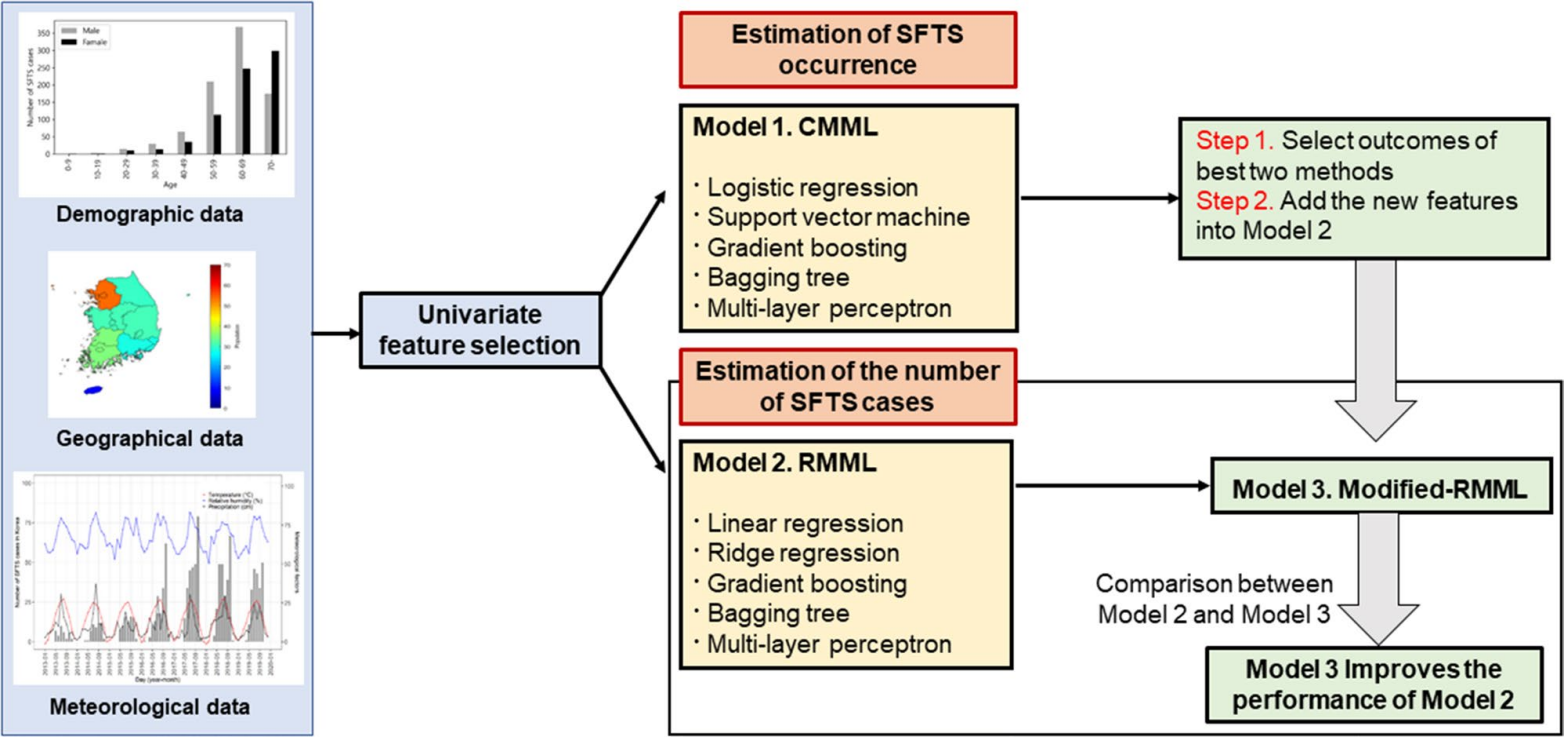
Schematic diagram predicting monthly occurrence and number of severe fever with thrombocytopenia syndrome cases.

The study approach for the statistical analysis of the three models is summarized in Fig. 6.

First, we estimated the geographical area of SFTS occurrence. A classification model in machine learning (CMML) was employed to identify the occurrence of an SFTS outbreak, which was defined as Model 1. Second, we estimated the number of SFTS cases by geographical area. The regression model in machine learning (RMML) was employed and was defined as Model 2. The machine learning methods for CMML and RMML are summarized in Supplementary Table S6. Moreover, we developed an additional model extending from the RMML by adding features obtained from the results of the CMML, which was defined as Model 3. Finally, the performance of all models was compared. The training and test datasets used epidemiological data from 2016 to 2018 and 2019, respectively.

### Data collection

Epidemiological data were categorized by demographic, geographic, and meteorological characteristics. The epidemic characteristics of SFTS cases in Korea from 2016 to 2019 were explored in Korea, as described in Supplementary Table S2. The regions in Korea were grouped into seven geographical areas: the Seoul Metropolitan Area (Seoul, Gyeonggi, Incheon), which is named as the capital area, and Chungcheong area (Daejeon, Chungnam, Chungbuk), Gyeongbuk area (Daegu, Gyeongbuk), Gyeongnam area (Busan, Ulsan, Gyeongnam), Honam area (Gwangju, Jeonnam, Jeonbuk), Gangwon-do, and Jeju. In addition, the non-capital area was defined as the six geographical areas, excluding the capital area.

Epidemiological data regarding SFTS incidence and deaths reported in Korea were provided by the Korea Disease Control and Prevention Agency^18^. Meteorological data, including the average maximum temperature, average minimum temperature, average temperature, average relative humidity, precipitation, and average percentage of sunshine, were provided by the Korea Meteorological Administration^20^. The geographical distribution data of the population, land area, forest area, rate of people aged 65 years and over, farm area, and number of farmers were provided by the Korean Statistical Information Service^21^. The geographical data relative to visitors to national parks were provided by the Korean National Park Service^22^.

### Feature selection

Overall, 15 features of epidemiological data are presented in Supplementary Table S2. Feature selection was conducted to select optimally predictive feature subsets for the target outcome.

Reducing the number of features results in improving the accuracy of the estimator or reducing the computational costs^23^. Univariate feature selection is a method of selecting important features based on statistical analysis, such as Chi-squared test, F test, and Analysis of variance (ANOVA). Feature selection was conducted according to the outcomes of SFTS occurrence and the number of SFTS cases using *F*-value of ANOVA. Feature selection was conducted according to the outcomes of SFTS and SFTS cases. Finally, we selected all features that were identified as important, in either the classification model or regression model, defined as being statistically significant, with a *P*-value < 0.05. The score for feature *I* was calculated as score $=-{\mathrm{log}}_{10}{p}_{i}/M$, where ${p}_{i}$ was the *P*-value of feature *i* and *M* was the maximum value of $\left({-\mathrm{log}}_{10}{p}_{i}\right)$ for all features. These important features obtained from the univariate selection were used to estimate the occurrence of SFTS or the number of SFTS cases.

### Estimation of severe fever with thrombocytopenia syndrome (SFTS) occurrence

The estimation of SFTS occurrence was conducted for seven geographical areas using epidemiological data based on environmental factors. Categorical variables (i.e., occurrence or non-occurrence of SFTS) were defined as SFTS occurrence if the monthly number of SFTS cases (*I*) in a geographical area was larger than 3; otherwise, SFTS was not observed. We employed five machine learning methods (CMML): logistic regression, support vector machine, gradient boosting, bagging tree, and multi-layer perceptron^19,24–27^. Here, the sample size of the train data was small in the present study (i.e., *n* = 252). We didn’t do cross-validation because the cross-validation can lead to a large error in predictive accuracy and reduce the reliability of prediction which is described in^28^. Moreover, when the data using Gradient-boosting and Bagging tree was learned, we conducted 100 simulations from 90% random sample. Afterward, the 95% confidence intervals (95% CIs) of 100 simulations of training and test data were obtained. To evaluate the performance of the machine learning methods, three measurements of accuracy, F1-score, and AUC were compared.

### Estimation of the number of SFTS cases

The monthly number of SFTS cases was estimated for seven geographical areas using linear regression, ridge regression, gradient boosting regression (GBR), bagging tree regression (BTR), and multi-layer perceptron regression (MLPR). Herein, using the features obtained from univariate feature selection showing *P*-value < 0.05, we introduced two regression models. The RMML is a well-known model described in previous studies^19,26,27,29,30^.

Second, we introduced a novel model called the modified-RMML (modified-RMML). The modified-RMML was developed by adding new features from the results of CMML to RMML. In other words, the CMML estimates the occurrence of SFTS in seven geographical areas. Subsequently, we selected the two best methods with the highest accuracy among the five machine learning methods. The binary outcomes (occurrence or non-occurrence) of the best two methods were added to the RMML as new features. We then tested whether the modified-RMML could improve accuracy by comparing the performance of RMML and the modified-RMML. Finally, the feature importance for all selected features was compared using Gini importance^31,32^, which was measured by averaging the impurity-based feature importance of each tree. An important feature is interpreted as one that is used more often in the split points of a tree.

### Ethical considerations

In the present study, we analyzed data that are publicly available in^18,20–22^. The publicly available data with no personally identifiable information does not require ethical approval.

## Supplementary Information

Supplementary Information.

## Data Availability

All datasets of epidemiological characteristics, including demographic, geographic, and meteorological data, are summarized in Supplementary Table S2. The data and code are available at https://github.com/giphil/SFTS_ML_Korea.
