# Supplementary material for: Estimating severe fever with thrombocytopenia syndrome transmission using machine learning methods in South Korea
Source: Sci Rep. 2021 Nov 8;11:21831. doi: 10.1038/s41598-021-01361-9 (PMC8575988; doi:10.1038/s41598-021-01361-9)
Supplement: Supplementary file 1 — Supplementary Information. [file 41598_2021_1361_MOESM1_ESM.pdf]

Table S1. Demographic characteristics of severe fever with thrombocytopenia syndrome cases in seven geographical areas from 2013 to 2019

Region	2013	2014	2015	2016	2017	2018	2019
Seoul Metropolitan area	1	13	11	47	69	62	54
Chungcheong area	2	5	7	23	46	38	35
Gyeongbuk area	11	20	14	29	44	41	31
Gyeongnam area	8	5	12	16	23	39	28
Honam area	5	1	11	13	30	29	35
Gangwon	3	4	15	29	39	35	30
Jeju	6	7	9	8	21	15	9

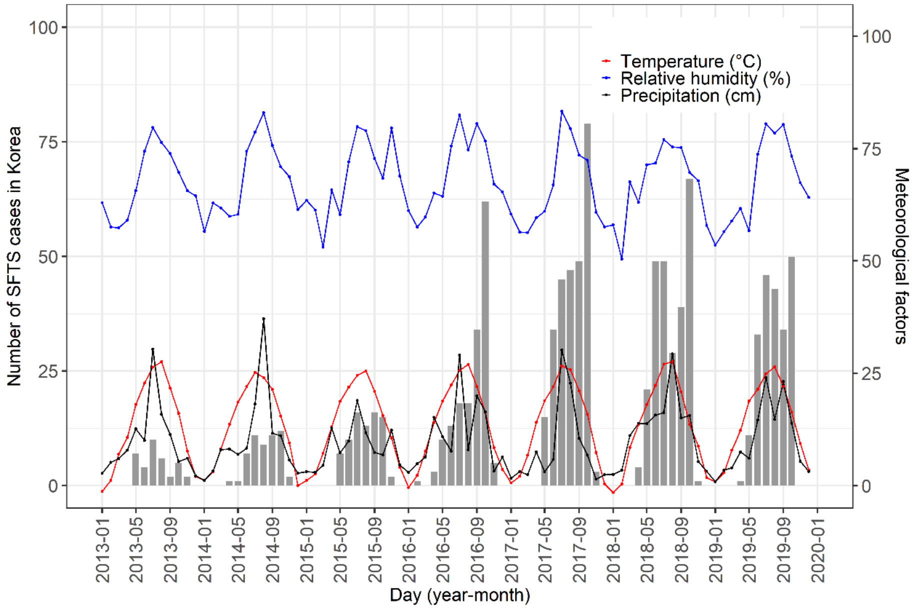

Figure S1. Number of severe fever with thrombocytopenia syndrome cases and monthly climate factors from 2013 to 2019 in Korea.

Table S2. Epidemiological characteristics of severe fever with thrombocytopenia syndrome cases in Korea from 2016 to 2019

Characteristics		Korea	S.M.*	Chungcheong	Gyeongbuk	Gyeongnam	Honam	Gangwon	Jeju
Demographic category	Total infected cases	913	232	137	145	106	107	133	53
	Male/female (%)	100	122	72.4	117	92.3	56.3	117	138
	Total deaths	160	40	28	27	16	25	17	7
	Population (thousand)	51,232	25,628	5,300	5,122	7,906	5,107	1,520	647
Geographic category	Land area (km^2)	99,900	11,855	16,175	19,916	12,371	20,906	16,828	1,850
	Forest (km^2)	63,549 (63,464, 63,923)	5,885 (5,868, 5,911)	9,315 (9,302, 9,337)	14,063 (14,050, 14,081)	8,012 (8,000, 8,029)	11,635 (11,617, 11,661)	13,769 (13,760, 13,782)	870 (868, 872)
	Rate of people aged 65 years and over (%)	0.154 (0.115, 0.192)	0.124 (0.115, 0.133)	0.147 (0.139, 0.156)	0.166 (0.155, 0.177)	0.139 (0.128, 0.150)	0.177 (0.169, 0.185)	0.181 (0.170, 0.192)	0.142 (0.139, 0.147)
	Farm (%)	0.060 (0.030, 0.093)	0.041 (0.040, 0.042)	0.065 (0.065, 0.065)	0.092 (0.091, 0.093)	0.046 (0.045, 0.046)	0.080 (0.079, 0.081)	0.065 (0.064, 0.067)	0.030 (0.030, 0.030)
	Rate of farmers (%)	0.138 (0.079, 0.205)	0.108 (0.104, 0.112)	0.150 (0.146, 0.154)	0.203 (0.200, 0.205)	0.101 (0.099, 0.102)	0.174 (0.172, 0.177)*	0.150 (0.146, 0.157)	0.081 (0.079, 0.081)
	Altitude (m)	178.629	79.233	162.667	173.500	111.667	156.333	525.000	42.000
	Mountain visitors (thousand)	2,749	481	288	533	222	668	474	79
Meteorological category	Average temperature (°C/month)	13.797 (-4.286, 28.450)	12.750 (−4.267, 28.100)	12.834 (−3.326, 27.868)	13.761 (−1.605, 27.570)	14.693 (0.869, 27.476)	13.976 (−0.871, 27.449)	11.675 (−4.286, 25.514)	16.891 (5.400, 28.450)
	Average maximum temperature	18.882 (0.020, 33.022)	17.779 (0.020, 32.607)	18.624 (1.650, 33.022)	19.404 (3.320, 32.830)	19.316 (5.419, 31.574)	19.727 (4.451, 32.776)	17.221 (0.971, 30.521)	20.105 (8.250, 31.700)

(°C/month)								
Average minimum temperature (°C/month)	9.401 (−9.036, 26.150)	8.482 (−7.873, 24.513)	7.705 (−7.709, 23.638)	8.846 (−5.915, 23.810)	10.780 (−3.033, 24.386)	9.129 (−5.245, 23.522)	6.731 (−9.036, 22.007)	14.131 (2.600, 26.150)
Average relative humidity (%/month)	67.605 (46.833, 91.000)	63.974 (49.600, 82.133)	68.87 (53.600, 85.578)	66.754 (50.250, 80.800)	67.717 (46.833, 86.833)	67.508 (47.441, 83.872)	65.292 (47.714, 82.000)	73.115 (59.500, 91.000)
Precipitation (mm/month)	106.076 (0.420, 529.580)	88.719 (0.420, 529.580)	94.734 (2.087, 452.851)	93.454 (4.125, 358.030)	115.933 (0.755, 337.436)	105.814 (1.743, 316.667)	99.561 (4.679, 436.854)	144.314 (12.950, 502.750)
Average percentage of sunshine (%/month)	53.642 (23.110, 72.708)	58.279 (28.641, 70.837)	54.422 (28.342, 68.142)	53.796 (29.475, 67.931)	56.957 (34.131, 72.708)	53.344 (30.150, 68.332)	53.475 (31.709, 71.070)	45.223 (23.110, 65.290)

Number in parentheses represent the minimum and maximum values.
S.M.* represents Seoul Metropolitan.

Table S3. Univariate feature selection to predict severe fever with thrombocytopenia syndrome (SFTS) occurrence and number of SFTS cases

**Features**	**Occurrence**			**SFTS case**		
	***F*-value**	***P*-value**	**Scores**	***F*-value**	***P*-value**	**Scores***
Month	18.071	<0.01	4.474	20.097	<0.01	4.892
Average temperature	124.251	<0.01	21.727	44.894	<0.01	9.625
Average maximum temperature	125.144	<0.01	21.844	47.977	<0.01	10.172
Average minimum temperature	122.29	<0.01	21.469	42.852	<0.01	9.258
Average relative humidity	82.345	<0.01	15.799	31.747	<0.01	7.195
Precipitation	48.191	<0.01	10.209	11.207	<0.01	3.006
Average percentage of Sunshine	16.243	<0.01	4.091	4.422	0.037	1.434
Land area	1.368	0.244	0.613	1.853	0.175	0.757
Forest	0.846	0.359	0.445	0.999	0.319	0.496
Population	0.401	0.527	0.278	8.068	<0.01	2.301
Rate of people aged 65 years and over	0.767	0.382	0.418	0.068	0.795	0.1
Farm	1.145	0.286	0.544	0.511	0.475	0.323
Rate of farmers	1.03	0.311	0.507	0.944	0.332	0.478
Altitude	<0.01	0.992	0.004	0.198	0.657	0.182
Mountain visitors	14.958	<0.01	3.819	33.924	<0.01	7.609

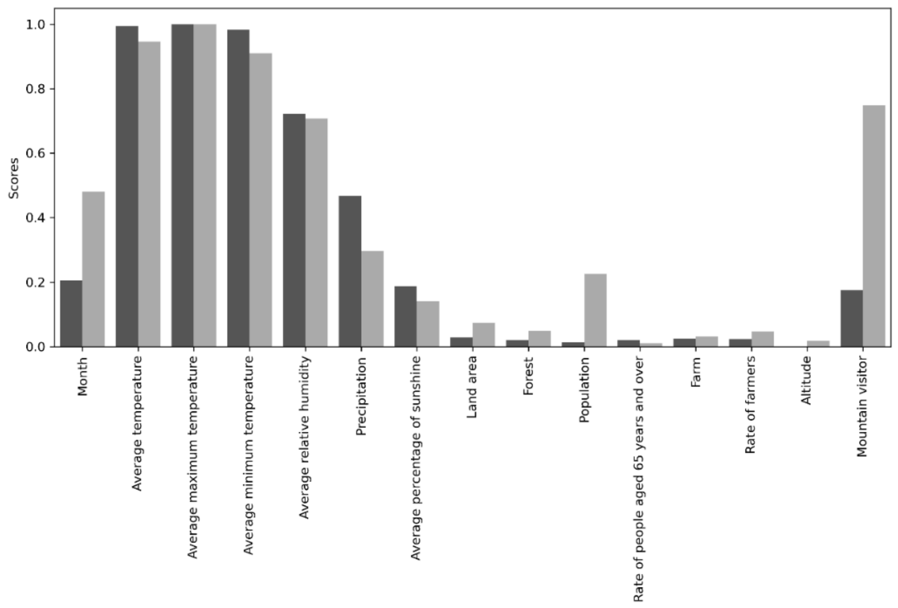

Figure S2. Comparison of the scores computed from the univariate feature selections according to severe fever with thrombocytopenia syndrome (SFTS) occurrence (black bars) and the number of SFTS case (gray bars), respectively, among the 15 features.

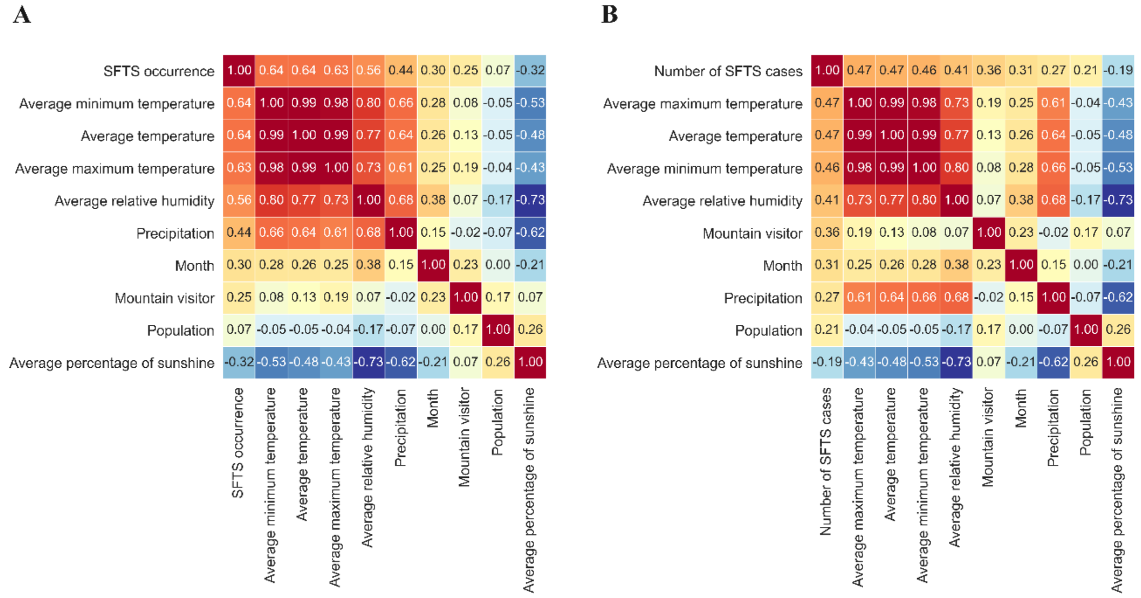

Figure S3. Correlation analysis of the nine features with (A) severe fever with thrombocytopenia syndrome (SFTS) occurrence and (B) the number of SFTS cases

Table S4. Performance of the estimation of severe fever with thrombocytopenia syndrome (SFTS) occurrence using the classification model in machine learning assessed using different thresholds

**Methods**	***z* = 1**		***z* = 2**		***z* = 3**		***z* = 4**		***z*= 5**	
	Accuracy	F1-score	Accuracy	F1-score	Accuracy	F1-score	Accuracy	F1-score	Accuracy	F1-score
LogR	0.893	0.892	0.881	0.861	0.917	0.885	0.869	0.792	0.845	0.606
SVM	0.929	0.929	0.893	0.873	0.940	0.923	0.952	0.926	0.881	0.722
GB	0.934 (0.905, 0.952)	0.934 (0.906, 0.952)	0.907 (0.893, 0.917)	0.885 (0.866, 0.899)	0.937 (0.929, 0.940)	0.915 (0.903, 0.921)	0.915 (0.899, 0.929)	0.859 (0.828, 0.885)	0.910 (0.893, 0.935)	0.829 (0.791, 0.880)
BT	0.940 (0.905, 0.964)	0.940 (0.906, 0.964)	0.913 (0.881, 0.952)	0.895 (0.857, 0.946)	0.936 (0.917, 0.952)	0.913 (0.885, 0.938)	0.926 (0.869, 0.964)	0.879 (0.770, 0.945)	0.890 (0.845, 0.929)	0.785 (0.684, 0.846)
MLP	0.869 (0.857, 0.881)	0.867 (0.857, 0.881)	0.893 (0.869, 0.905)	0.873 (0.849, 0.886)	0.940 (0.917, 0.952)	0.921 (0.892, 0.938)	0.894 (0.881, 0.905)	0.831 (0.808, 0.852)	0.866 (0.833, 0.887)	0.738 (0.640, 0.792)

z indicates the threshold value, which means that SFTS occurrence is determined if the monthly SFTS case in geographical area is larger than or equal to *z*. Number in parentheses represents the 95% confidence interval obtained from 100 simulations.

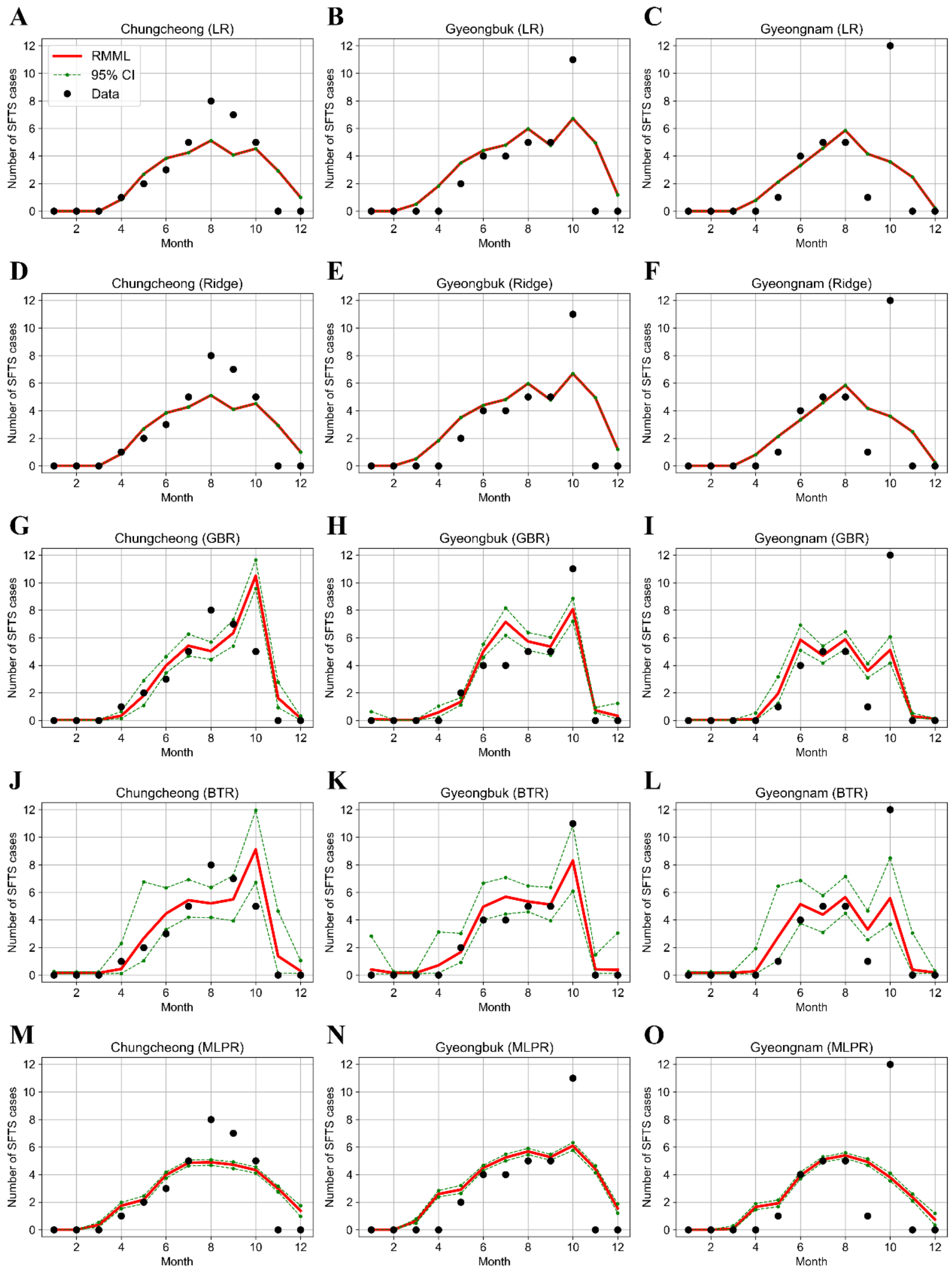

Figure S4. Estimation of the number of severe fever with thrombocytopenia syndrome (SFTS) cases in 2019 using five machine learning methods of the regression model in machine learning (RMML). The black dots represent the observed cases. The red solid lines indicate the estimated SFTS cases with 95% confidence interval shown in green dashed lines. The results of three geographical areas of Chungcheong, Gyeongbuk, and Gyeongnam are shown. Parentheses represent the machine learning methods of RMML.

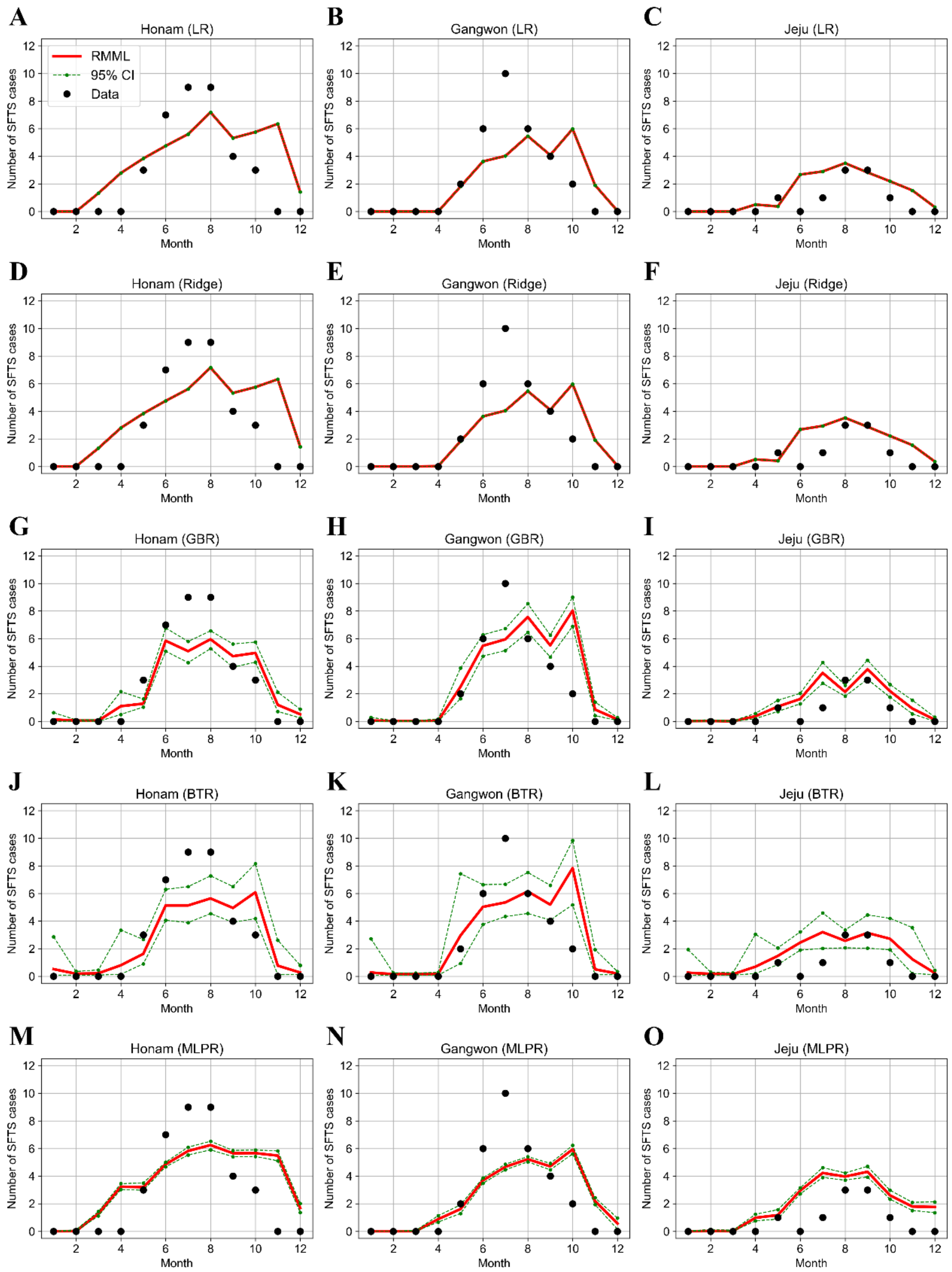

Figure S5. Estimation of the number of severe fever with thrombocytopenia syndrome (SFTS) cases in 2019 using five machine learning methods of the regression model in machine learning (RMML). The black dots represent the observed cases. The red solid lines indicate the estimated SFTS cases with 95% confidence intervals shown in green dashed lines. The results of three geographical areas of Honam, Gangwon, and Jeju are shown. Parentheses represent the machine learning methods of RMML.

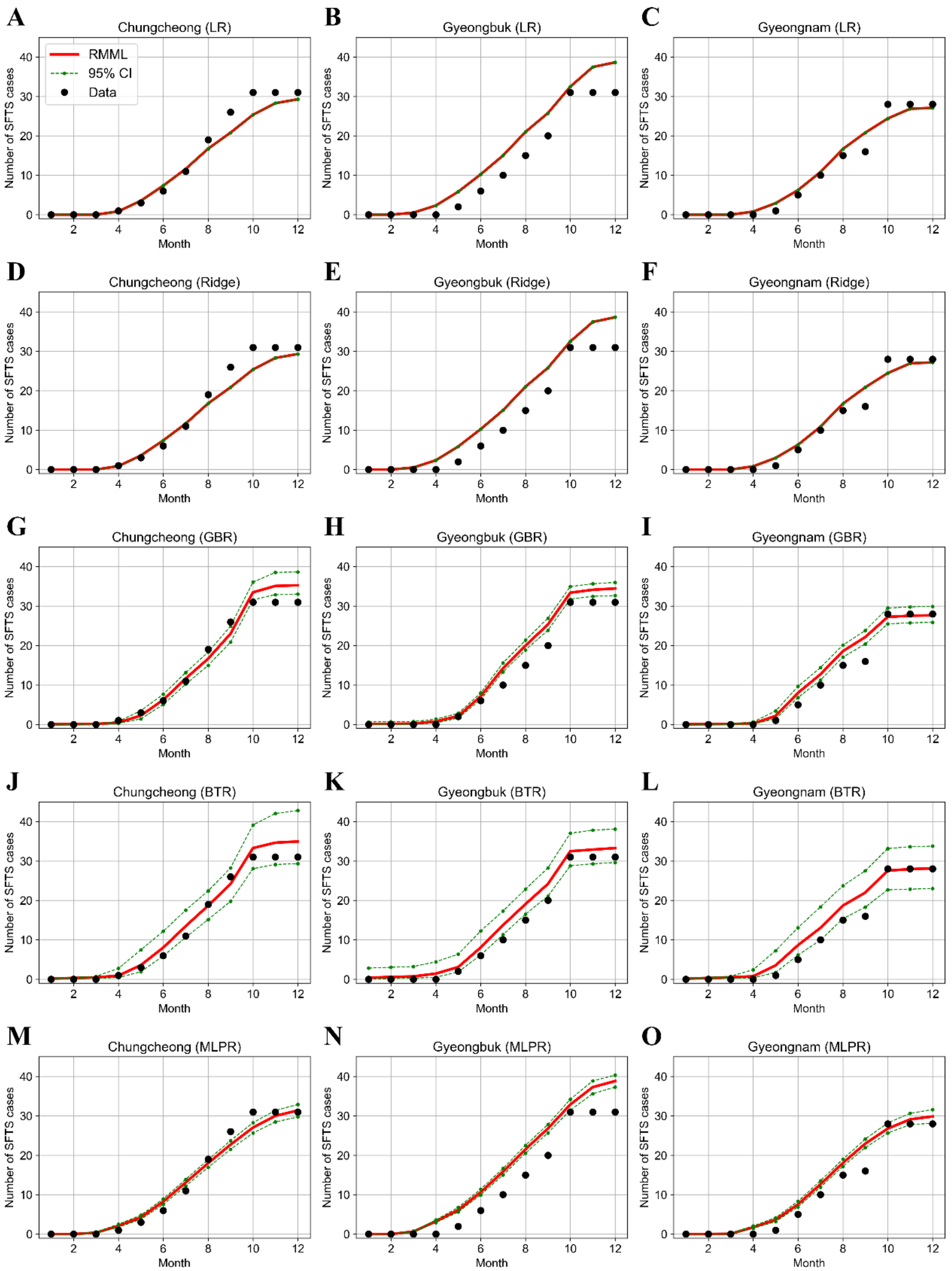

Figure S6. Estimation of the cumulative number of severe fever with thrombocytopenia syndrome (SFTS) cases in 2019 using five machine learning methods of the regression model in machine learning (RMML). The black dots represent the cumulative cases of data in three geographical areas of Chungcheong, Gyeongbuk and Gyeongnam. The red solid lines indicate the estimated values of the SFTS cumulative cases with 95% confidence intervals shown in green dashed lines. Parentheses represent the machine learning methods of RMML.

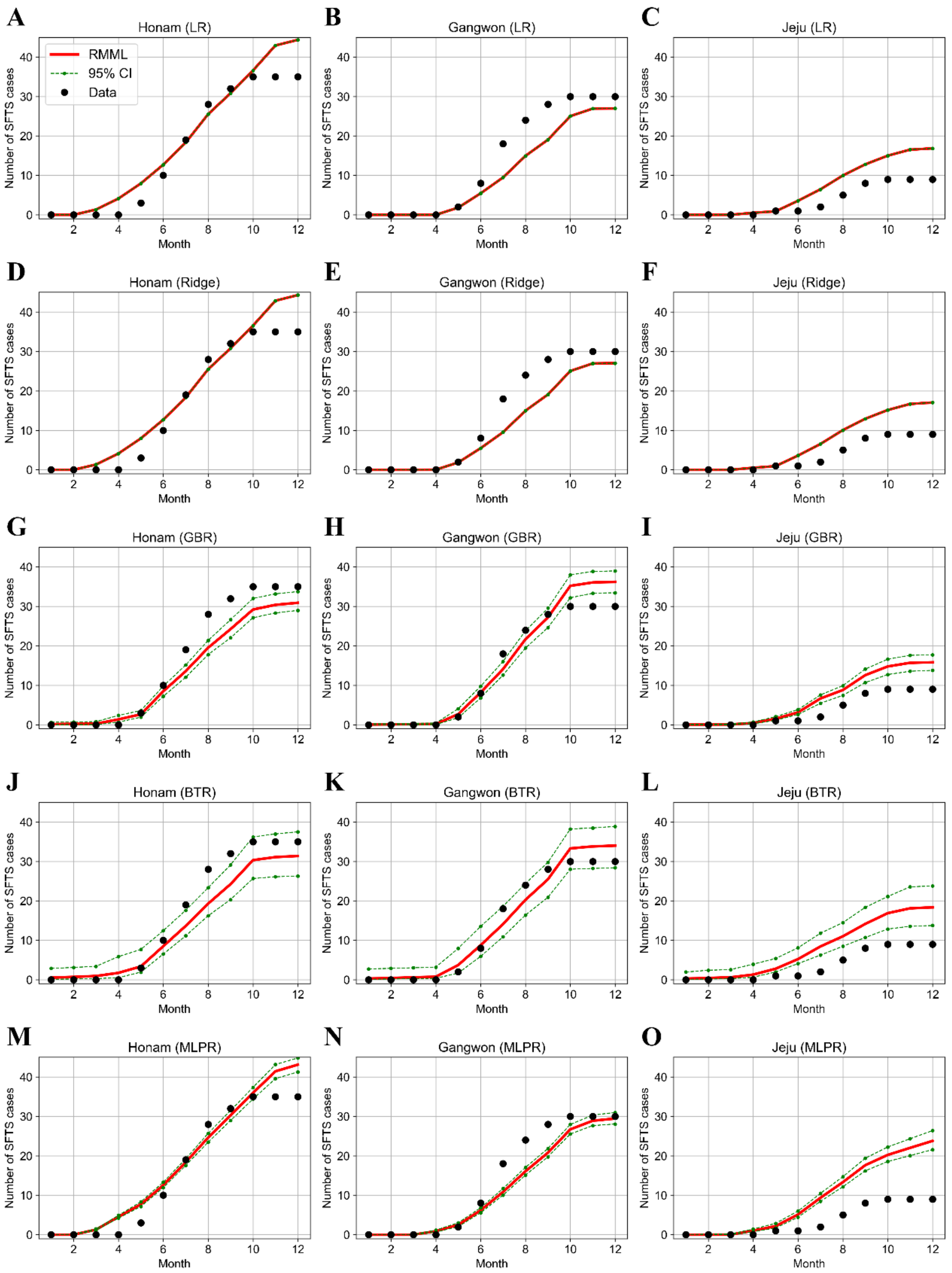

Figure S7. Estimation of the cumulative number of severe fever with thrombocytopenia syndrome (SFTS) cases in 2019 using five machine learning methods of the regression model in machine learning (RMML). The black dots represent the cumulative cases of data in three geographical areas of Honam, Gangwon, and Jeju. The red solid lines indicate the estimated values of the SFTS cumulative cases with 95% confidence intervals shown in green dashed lines. Parentheses represent the machine learning methods of RMML.

Table S5. Performance of the training and test data of severe fever with thrombocytopenia syndrome cases using the five modified regression models in machine learning (modified-RMML)

Methods	Modified-RMML			
	Train		Test	
	MSE	R^2	MSE	R^2
LR	6.558 (−40.6)	0.636 (64.5)	4.175 (−35.3)	0.687 (33.0)
Ridge	6.562 (−40.6)	0.636 (64.6)	4.162 (−35.5)	0.689 (33.2)
GBR	0.187 (−54.8)	0.990 (1.29)	3.127 (−14.2)	0.766 (5.31)
BTR	1.373 (−46.6)	0.924 (7.75)	2.937 (−12.6)	0.780 (4.24)
MLPR	2.329 (−79.2)	0.871 (130)	3.190 (−52.2)	0.761 (52.0)

Parentheses represent the percentage decrease of MSE from the modified-RMML compared to the RMML.

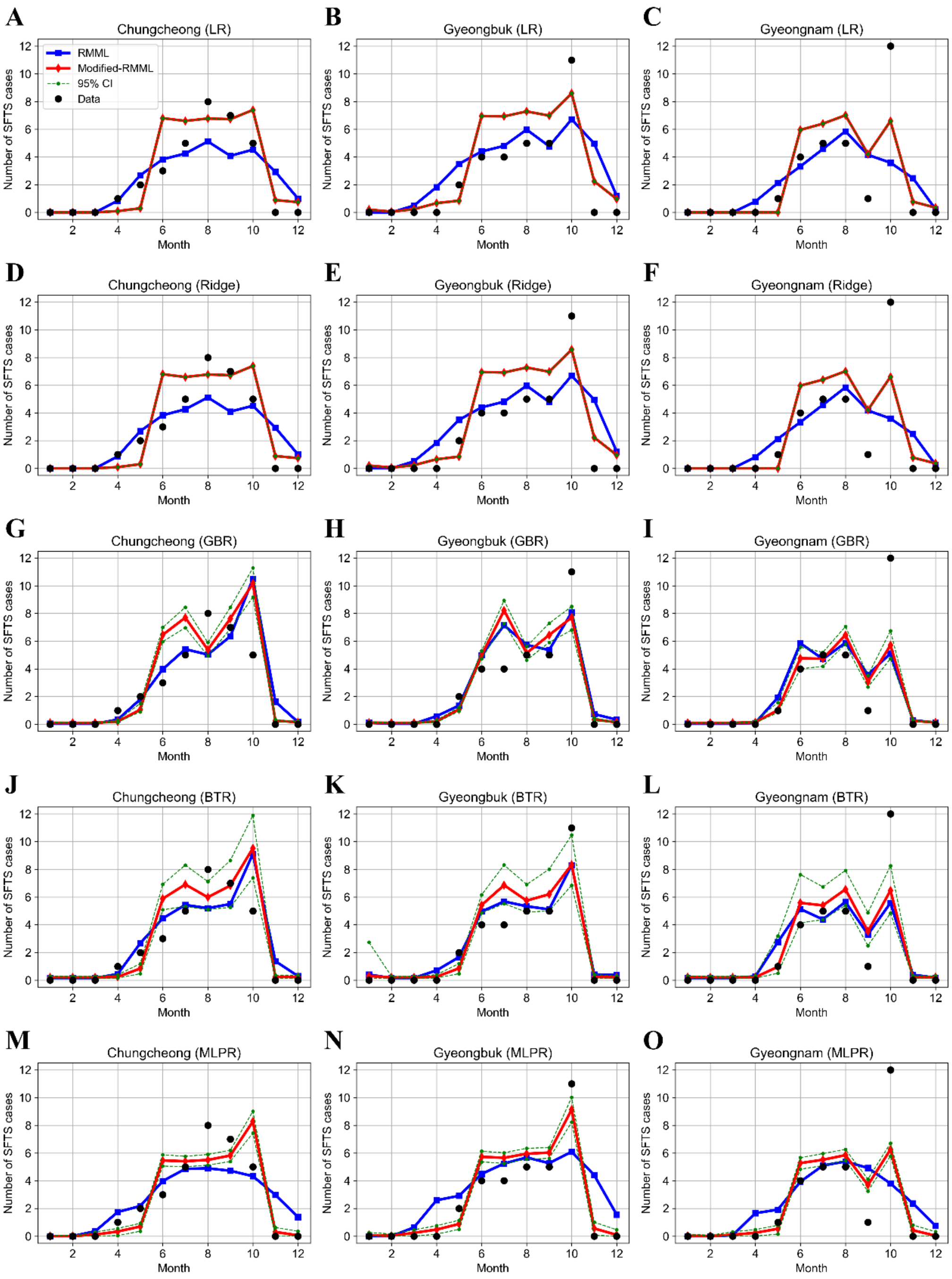

Figure S8. Comparison of the results of the regression model in machine learning (RMML) and modified RMML to estimate the number of severe fever with thrombocytopenia syndrome (SFTS) cases in 2019. The black dots represent the observed cases of data in three geographical areas of Chungcheong, Gyeongbuk and Gyeongnam. The blue sold lines and red solid lines indicate the estimated SFTS cases from RMML and modified RMML, respectively. The 95% confidence intervals of the modified RMML are shown in green dashed lines. Parentheses represent the five machine learning methods.

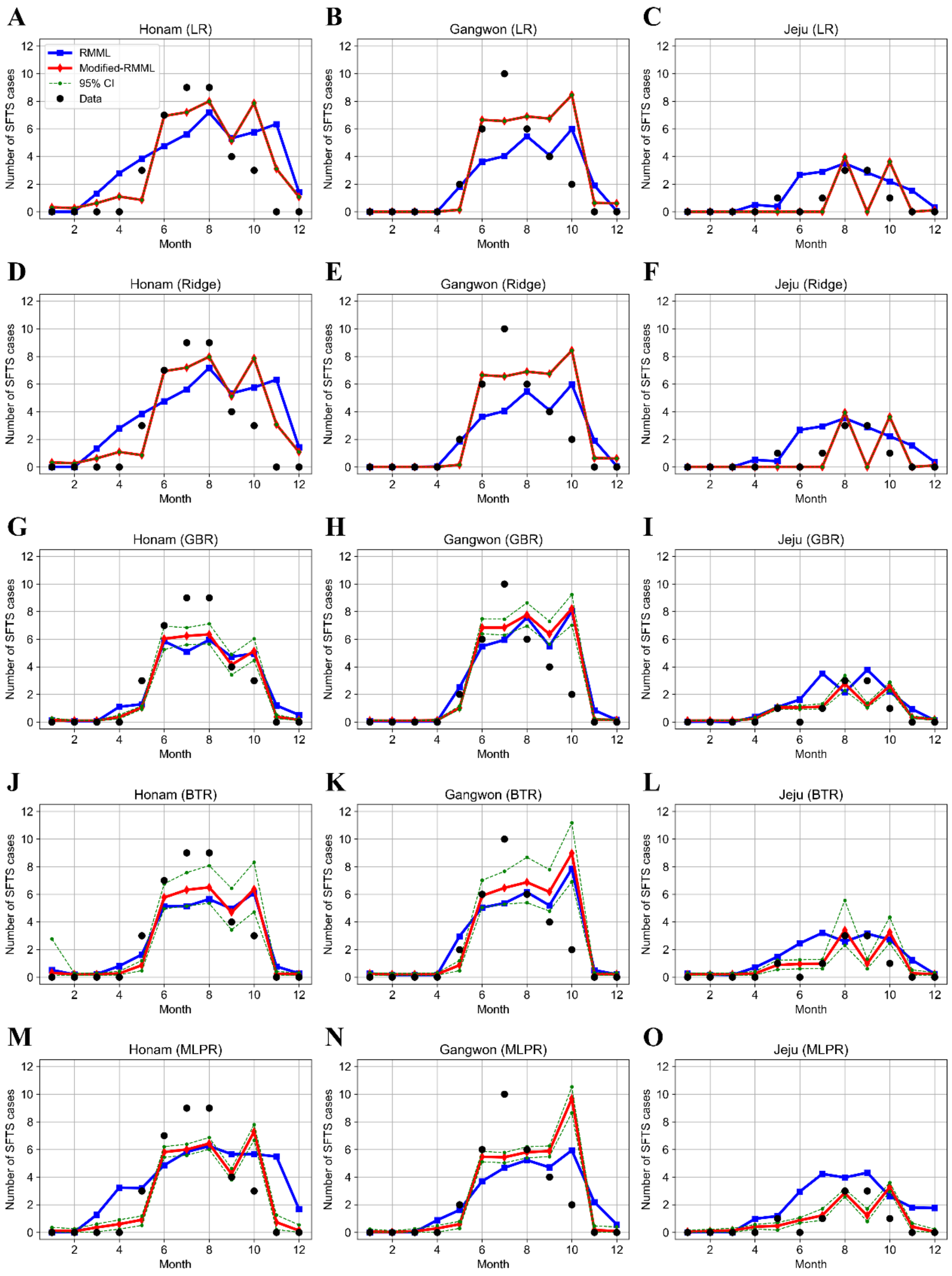

Figure S9. Comparison of the results of the regression model in machine learning (RMML) and modified RMML to estimate the number of severe fever with thrombocytopenia syndrome (SFTS) cases in 2019. The black dots represent the observed cases of data in three geographical areas of Honam, Gangwon, and Jeju. The blue sold lines and red solid lines indicate the estimated SFTS cases from RMML and modified RMML, respectively. The 95% confidence intervals of the modified RMML are shown in green dashed lines. Parentheses represent the five machine learning methods.

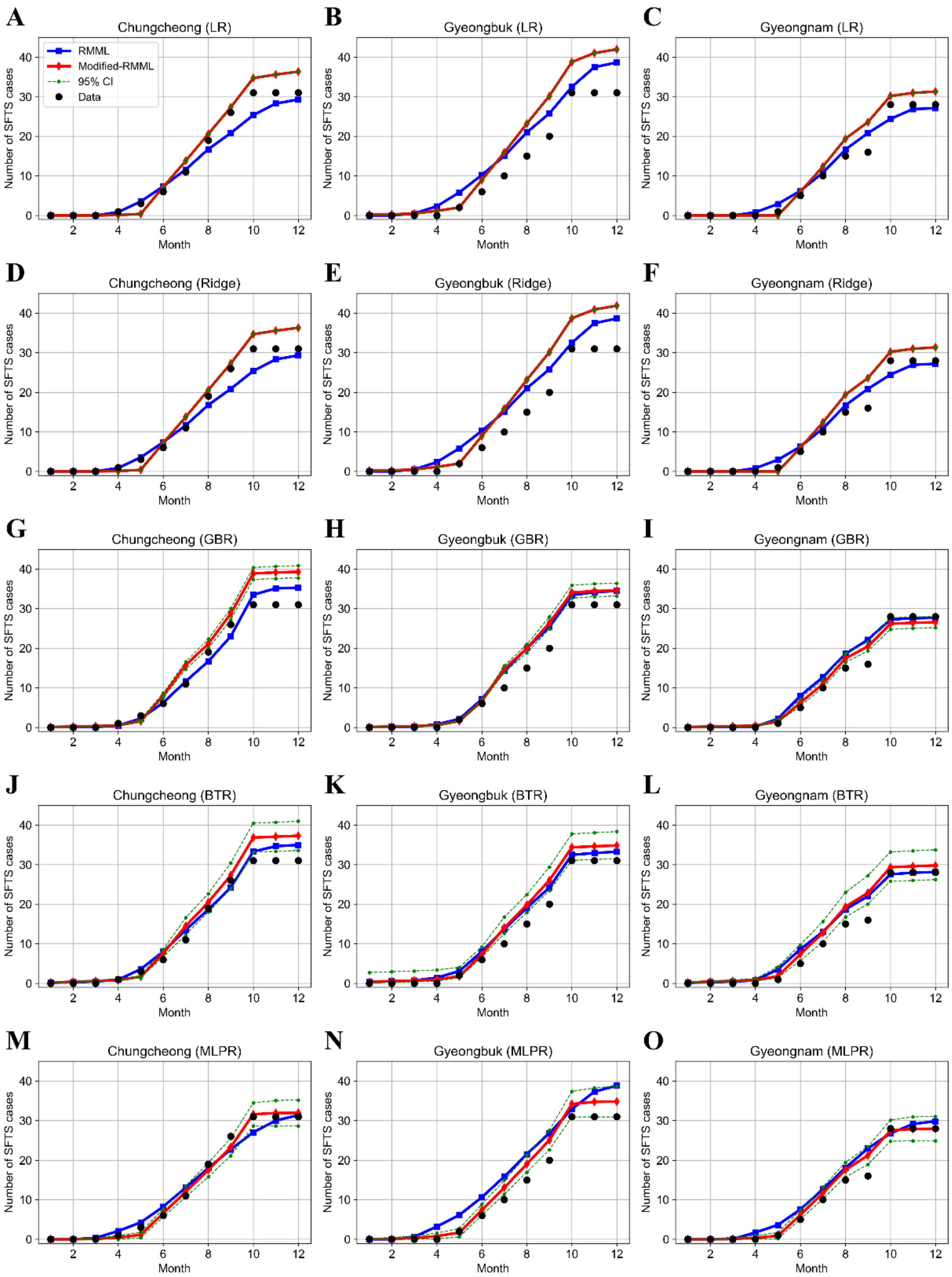

Figure S10. Comparison of the results of the regression model in machine learning (RMML) and modified RMML to estimate the cumulative number of severe fever with thrombocytopenia syndrome (SFTS) cases in 2019. The black dots represent the cumulative cases of data in three geographical areas of Chungcheong, Gyeongbuk, and Gyeongnam. The blue sold lines and red solid lines indicate the estimated values of the SFTS cumulative cases from RMML and modified RMML, respectively. The 95% confidence intervals of the modified RMML are shown in green dashed lines. Parentheses represent the five machine learning methods.

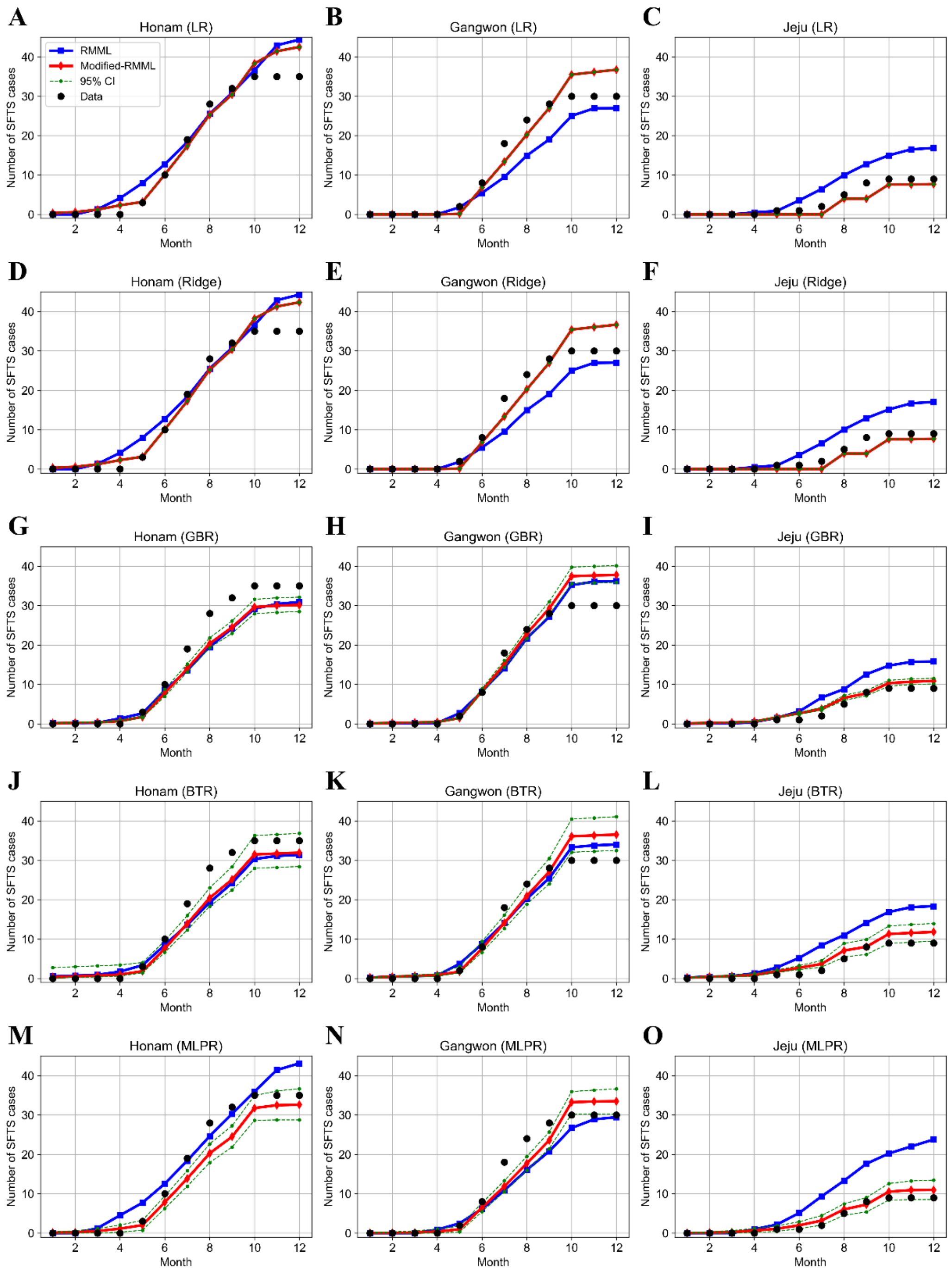

Figure S11. Comparison of the results of the regression model in machine learning (RMML) and modified RMML to estimate the cumulative number of severe fever with thrombocytopenia syndrome (SFTS) cases in 2019. The black dots represent the cumulative cases of data in three geographical areas of Honam, Gangwon, and Jeju. The blue sold lines and red solid lines indicate the estimated values of the SFTS cumulative cases from RMML and modified RMML, respectively. The 95% confidence intervals of the modified RMML are shown in green dashed lines. Parentheses represent the five machine learning methods.

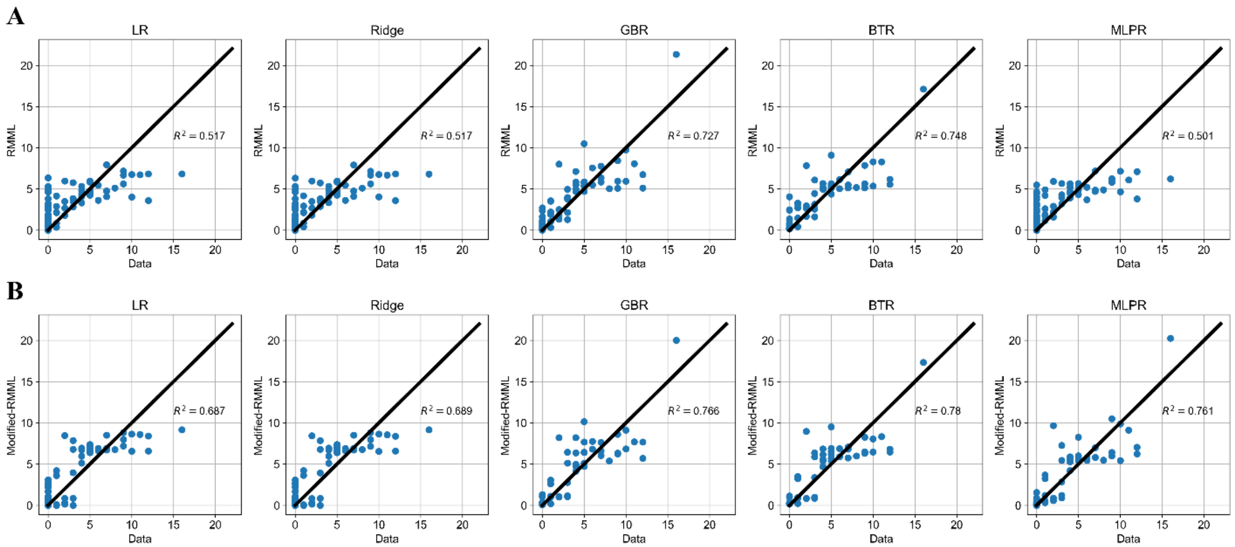

Figure S12. Comparison between observed and estimated number of severe fever with thrombocytopenia syndrome cases in 2019, using five machine learning methods of the regression model in machine learning (RMML) (A) and modified RMML (B), respectively.

Table S6. Description of a classification model in machine learning and a regression model in machine learning

Method	Description	Model	Ref.
Logistic regression (LogR)	A predictive regression analysis when the dependent variable is binary	Classification	[24]
Support vector machine (SVM)	A classifier that is defined by an optimal linear separation of the hyperplane that discriminates between labels	Classification	[25]
Gradient boosting (GB) and gradient boosting regression (GBR)	A machine learning technique for regression and classification, which produces a prediction model in the form of an ensemble of weak prediction models, typically decision trees. GB is used for the classification model, and GBR is used for the regression model.	Classification, regression	[26]
Bagging tree (BT) and bagging tree regression (BTR)	Reduces the variance of a decision tree and creates several subsets of data from the training sample chosen randomly with replacement. BT is used for the classification model, and BTR is used for the regression model.	Classification, regression	[19]
Linear regression (LR)	Linear approach to model the association between a scalar response and one or more explanatory variables. The case of one explanatory variable is called simple LR; for more than one, the process is called multiple LR.	Regression	[28]
Ridge regression (Ridge)	Ridge is a popular parameter estimation method used to address collinearity problems frequently arising in multiple LR.	Regression	[29]
Multi-layer perceptron (MLP) and multi-layer perceptron regression (MLPR)	The MLP is a class of feed-forward artificial neural network (ANN). The term MLP is used ambiguously, sometimes loosely to any feed-forward ANN, and sometimes strictly refers to networks composed of multiple layers of perceptron. MLP is used for the classification model, and MLPR is used for the regression model.	Classification, regression	[27]

Ref. indicates the references.
